# Correlation of serum cytokine levels with disease severity in psoriasis: A physiological insight into inflammatory markers

**DOI:** 10.6026/973206300212803

**Published:** 2025-08-31

**Authors:** Raghav Gupta, Devpriya Shukla, Amit Saxena, Sanjay Prasad

**Affiliations:** 1 Department of Dermatology, Bundelkhand Medical College, Sagar, Madhya Pradesh, India; 2 Department of General Medicine, Bundelkhand Medical College, Sagar, Madhya Pradesh, India; 3 Department of Psychiatry, Bundelkhand Medical College, Sagar, Madhya Pradesh, India

**Keywords:** Psoriasis, cytokines, inflammation, TNF-α, IL-6, IL-17, PASI score, biomarkers, disease severity

## Abstract

Psoriasis is a chronic immune-mediated skin disorder in which systemic inflammation and cytokine dysregulation play central roles. Identifying correlations between cytokine levels and disease severity may aid in establishing reliable biomarkers. In this cross-sectional study, 60 psoriasis patients and 30 healthy controls were evaluated; PASI scores were recorded and serum TNF-α, IL-6 and IL-17 levels was measured using ELISA. All three cytokines were significantly elevated in patients and showed strong positive correlations with PASI scores. Elevated TNF-α, IL-6 and IL-17 may serve as biomarkers of disease activity and potential therapeutic targets in psoriasis management.

## Background:

Psoriasis is a chronic immune-mediated inflammatory skin disorder affecting about 2-3% of the global population and typically manifests as persistent red, scaly and thickened plaque-type lesions [1]. Beyond cutaneous involvement, psoriasis is associated with systemic comorbidities such as psoriatic arthritis, cardiovascular disease and metabolic syndrome, reflecting its systemic inflammatory nature [2]. Genetic predisposition, environmental factors and immune dysregulation interact in a complex manner at the core of psoriasis pathogenesis. Central to this process is the activation of T-helper (Th) subsets, particularly Th1 and Th17, which secrete cytokines including tumor necrosis factor-alpha (TNF-α), interleukin-6 (IL-6) and interleukin-17 (IL-17), driving keratinocyte hyperproliferation and chronic inflammation [3]. These cytokines not only sustain cutaneous lesions but also contribute to systemic inflammatory burden in psoriasis patients [4]. Serum cytokine analysis provides insight into the inflammatory milieu and serves as a surrogate marker of disease activity. Studies have demonstrated that patients with active psoriasis exhibit elevated TNF-α, IL-6 and IL-17 levels, which positively correlate with Psoriasis Area and Severity Index (PASI) scores [5]. However, the precise relationship between circulating cytokines and clinical severity remains an area of on-going research. Cytokine profiling has shown potential for understanding disease mechanisms and guiding personalized therapy in psoriasis. Biologics targeting TNF-α (*e.g.*, etanercept, adalimumab), IL-17 (*e.g.*, secukinumab) and IL-23 have demonstrated marked efficacy, highlighting the pivotal role of these molecules in disease pathology [6]. Monitoring cytokine levels may help predict disease flares, assess treatment response and enable tailored immunomodulatory therapy to improve outcomes while minimizing adverse effects [7]. IL-6 is a multifunctional cytokine with both pro- and anti-inflammatory properties, regulating T-cell differentiation, B-cell activation and acute-phase responses. It promotes keratinocyte proliferation and survival, contributing to psoriatic epidermal hyperplasia [8]. Elevated IL-6 has been linked not only to disease severity but also to systemic comorbidities such as fatigue, cardiovascular risk and insulin resistance in psoriasis [9]. IL-17, primarily secreted by Th17 cells, plays a central role in psoriasis pathogenesis by directly stimulating keratinocytes, inducing antimicrobial peptide production and recruiting neutrophils through chemokine release [10]. Serum and lesional IL-17 concentrations correlate strongly with PASI and IL-17 inhibition results in significant clinical improvement, underscoring its role as both a biomarker and therapeutic target. Therefore, it is of interest to report the correlation between serum levels of TNF-α, IL-6 and IL-17 with psoriasis severity, providing insights into their utility as immunological markers of disease activity.

## Materials and Methods:

A total of 60 patients diagnosed clinically with chronic plaque psoriasis were included in the study. Diagnosis was confirmed based on characteristic clinical features, with or without histopathological confirmation when necessary. Patients aged 18-65 years, not on systemic immunosuppressive or biological therapy for at least four weeks, were included. Individuals with other inflammatory or autoimmune disorders, active infections, or malignancies were excluded. Thirty age- and sex-matched healthy individuals served as the control group. Detailed clinical evaluation was conducted and disease severity was assessed using the Psoriasis Area and Severity Index (PASI) score. Blood samples (5 mL) were collected aseptically from both patient and control groups. Serum was separated by centrifugation at 3000 rpm for 10 minutes and stored at -80°C until analysis. Quantitative measurement of serum TNF-α, IL-6 and IL-17 levels was performed using commercially available enzyme-linked immunosorbent assay (ELISA) kits (BioLegend®, USA), following the manufacturers protocol. All assays were conducted in duplicate to ensure accuracy and reproducibility. Statistical analysis was conducted using SPSS version 26.0 (IBM Corp., USA). Descriptive data were expressed as mean ± standard deviation (SD). Differences in cytokine levels between patients and controls were assessed using the independent t-test. Pearson's correlation coefficient was applied to evaluate the relationship between cytokine levels and PASI scores. A p-value of <0.05 was considered statistically significant.

## Results:

A total of 60 patients with chronic plaque psoriasis (38 males and 22 females) and 30 age- and sex-matched healthy controls were included in the study. The mean age of the psoriasis group was 42.6 ± 10.2 years, while that of the control group was 41.3 ± 9.5 years, with no statistically significant difference (p = 0.48). The average duration of psoriasis was 6.8 ± 3.1 years. The mean PASI score among patients was 18.2 ± 5.4, indicating moderate to severe disease activity (Table 1 - see PDF). Serum levels of TNF-α, IL-6 and IL-17 were significantly higher in psoriatic patients compared to healthy controls. Mean TNF-α levels were 36.8 ± 7.2 pg/mL in patients versus 12.4 ± 3.1 pg/mL in controls (p < 0.001). IL-6 levels averaged 29.7 ± 6.5 pg/mL in the psoriasis group compared to 9.8 ± 2.9 pg/mL in controls (p < 0.001). Similarly, IL-17 levels were elevated in patients (42.3 ± 8.1 pg/mL) compared to controls (11.3 ± 2.5 pg/mL) (p < 0.001), as shown in Table 2 (see PDF). To explore the relationship between cytokine levels and disease severity, Pearson's correlation analysis was performed. A strong positive correlation was observed between PASI score and TNF-α (r = 0.71), IL-6 (r = 0.65) and IL-17 (r = 0.76), all of which were statistically significant (p < 0.001) (Table 3 - see PDF). Additionally, subgroup analysis based on PASI score severity showed progressively higher cytokine levels in patients with more severe disease (Table 4 - see PDF). Patients were stratified into mild (PASI <10), moderate (PASI 10-20) and severe (PASI >20) groups. A stepwise increase in cytokine concentration was evident with increasing disease severity. These findings suggest a robust association between elevated inflammatory cytokines and the clinical severity of psoriasis, further reinforcing their role as potential biomarkers for disease monitoring.

## Discussion:

The findings of this study demonstrate a significant elevation in the serum levels of pro-inflammatory cytokines TNF-α, IL-6 and IL-17 in patients with chronic plaque psoriasis compared to healthy controls. Additionally, these cytokine levels showed a strong positive correlation with disease severity as measured by PASI scores, aligning with the current understanding of psoriasis as a systemic inflammatory disorder driven by immune dysregulation. TNF-α is a central cytokine in the inflammatory cascade and has long been implicated in the pathogenesis of psoriasis. It promotes the recruitment and activation of immune cells, as well as keratinocyte proliferation, contributing to plaque formation [1]. In our study, serum TNF-α levels were markedly elevated in psoriatic patients and correlated significantly with PASI scores, supporting its established role in disease progression and justifying the use of TNF inhibitors as effective therapeutic agents [2, 3]. IL-6 is another multifunctional cytokine that plays a pivotal role in T cell differentiation and acute-phase responses. Its overexpression in psoriatic skin leads to increased keratinocyte survival and impaired apoptosis, contributing to epidermal thickening [4]. Our findings are consistent with prior research reporting increased IL-6 levels in both lesional skin and systemic circulation of psoriatic patients [5]. Furthermore, IL-6 has been associated with the development of comorbidities such as insulin resistance and cardiovascular dysfunction in these patients, making it a potential marker for both cutaneous and systemic disease burden [6, 7]. IL-17, primarily secreted by Th17 cells, is now recognized as a key effector cytokine in psoriasis. It acts directly on keratinocytes, inducing the expression of antimicrobial peptides and neutrophil-attracting chemokines, thus sustaining inflammation [8]. In the current study, IL-17 showed the highest correlation with PASI scores among the cytokines analyzed, indicating its close association with disease severity. This is supported by multiple studies and the clinical success of IL-17-targeted biologics such as secukinumab and ixekizumab in managing moderate-to-severe psoriasis [9, 10]. The progressive increase in cytokine levels with increasing PASI severity groups further reinforces the concept that serum cytokine profiling can reflect clinical disease activity. These findings are in line with the results of Arora et al., who found a direct relationship between cytokine levels and clinical scores [11]. Additionally, our study supports the utility of cytokine measurements as potential biomarkers for monitoring disease and tailoring treatment regimens. Despite the valuable insights, several limitations must be acknowledged. The cross-sectional design restricts the ability to infer causality. Longitudinal studies are necessary to assess the dynamic changes in cytokine levels with treatment and disease remission. Moreover, other relevant cytokines such as IL-23, IFN-γ and IL-22 were not measured, which may have provided a more comprehensive understanding of the inflammatory network in psoriasis [12, 13]. The study also did not assess the impact of previous treatments on cytokine levels, which could have influenced the findings. Nevertheless, this study highlights the importance of systemic inflammation in psoriasis and provides physiological evidence of the association between elevated cytokines and disease severity. The consistent pattern of increased TNF-α, IL-6 and IL-17 levels supports the paradigm shift from viewing psoriasis as a purely cutaneous disease to recognizing it as a systemic immune-mediated disorder [14]. Future research should explore the integration of cytokine profiling into routine clinical practice for disease monitoring and personalized therapeutic decision-making [15].

## Conclusion:

A significant correlation between elevated serum levels of TNF-α, IL-6 and IL-17 with psoriasis severity. These cytokines may serve as reliable biomarkers for disease activity. Their monitoring could aid in personalized treatment strategies and predicting therapeutic response.
